# Clinical and genetic characteristics of hypophosphatasia in Chinese children

**DOI:** 10.1186/s13023-021-01798-1

**Published:** 2021-04-07

**Authors:** Meijuan Liu, Min Liu, Xuejun Liang, Di Wu, Wenjing Li, Chang Su, Bingyan Cao, Jiajia Chen, Chunxiu Gong

**Affiliations:** Department of Endocrinology, Genetics and Metabolism, Beijing Children’s Hospital, Capital Medical University, National Center for Children’s Health, Beijing, 100045 China

**Keywords:** Hypophosphatasia, *ALPL*, Mutations, China, Children

## Abstract

**Background:**

Hypophosphatasia (HPP) is a rare inherited disorder, which is caused by loss-of-function mutations in the *ALPL* gene. HPP is a heterogeneous disease that has a wide spectrum of phenotypes. Few studies were carried out in the Chinese population with HPP, especially in children.

**Methods:**

The clinical and genetic characteristics of 10 Chinese children with HPP who were referred to the Beijing Children’s Hospital were described. Previously reported HPP cases of children in China were also reviewed.

**Results:**

A total of 33 cases were identified, which included 2 perinatal lethal HPP, 10 infantile HPP, 10 childhood HPP, and 11 odonto HPP. The male-to-female ratio was 24:9. The average age at onset was 0.69 years (ranged from 2 h after birth to 14 years), while the average age at clinical diagnosis was 3.87 years (ranged from 2 h after birth to 19 years). Serum alkaline phosphatase (ALP) levels were significantly decreased in patients with perinatal lethal/infantile HPP when compared with those with the mild forms of HPP childhood/odonto HPP (*P* < 0.01). Although serum phosphate levels were not different (*P* > 0.05), serum calcium levels were elevated, and serum intact parathyroid hormone levels were decreased in patients with perinatal lethal/infantile HPP in comparison with those with the childhood/odonto HPP (*P* all < 0.01). Genetic analyses identified 40 mutations in 31 HPP cases, including 28 missense mutations, 9 frameshift mutations, 2 splice junction alterations, and 1 regulatory mutation. Of which, 5 novel mutations were identified in our present study: 2 frameshift mutations (p.Arg138GlyfsTer27, p.Leu511Profs*272); 2 missense mutations (p.Ala176Val, p.Phe268Leu), and 1 splice junction alteration (c.297+5G>A). Compound heterozygous mutations accounted for 80.6% of all variants. No mutational “hot-spot” was found. Most mutations of *ALPL* were located in exons 5, 7, 10, and 3. Notably, subjects that carrying single heterozygous mutations showed milder phenotypes of HPP, while subjects with nonsense mutations were associated with a severer phenotype.

**Conclusions:**

HPP is a rare disease with often delayed diagnosis, and the incidence of HPP in China may be seriously underestimated. The present study expands the phenotypic and genotypic spectrum and the understanding of HPP in Chinese children. These findings will be useful for clinical assessment and shorten the diagnosis time for pediatric HPP in China.

**Supplementary Information:**

The online version contains supplementary material available at 10.1186/s13023-021-01798-1.

## Background

Hypophosphatasia (HPP, OMIM: 146300, 241500, 241510), which was initially reported by Rathbun [1], is a rare inherited metabolic disorder. It is caused by the loss-of-function mutations in the *ALPL* gene (MIM 171760) that encodes the tissue-nonspecific isozyme of alkaline phosphatase (TNSALP), which is a homodimeric phosphohydrolase and abundantly expressed in the skeleton, developing teeth, liver, and kidney [2]. There are three well-known extracellular substrates of TNSALP, including inorganic pyrophosphate (PPi), pyridoxal-5-phosphate (PLP), and phosphoethanolamine (PEA) [3]. Among them, PPi is a potent inhibitor of mineralization [3]. Thus, the reduced TNSALP activity that due to the mutations in the *ALPL* gene results in the extracellular accumulation of PPi, which further leads to defective mineralization of bones and teeth.

Although HPP is characterized by impaired mineralization of bones and teeth, and reduced serum alkaline phosphatase (ALP) activity, the clinical spectrum of HPP is extremely variable. Based on the age of onset and the clinical symptoms, HPP is currently classified into six phenotypes: perinatal lethal, perinatal benign, infantile, childhood, adult, and odontohypophosphatasia (odonto-HPP) [2]. Both perinatal lethal and infantile were identified as the severe forms of HPP, whereas perinatal benign, childhood, adult, and odonto-HPP were defined as the mild forms of HPP [4–8]. It has been reported that the severe forms of HPP are usually recessively inherited, while the mild forms of HPP show both dominant and recessive inheritance [6]. At present, the diagnosis of HPP remains as before, relied on clinical indications, while the genetic sequencing of the *ALPL* gene is a useful technique for the precise diagnosis of HPP.

The *ALPL* gene is located on chromosome 1p36.1-p34 [9] and comprises 12 exons distributed over 70 kb [10]. As of May 2020, a total of 410 different *ALPL* mutations have been reported worldwide (http://www.sesep.uvsq.fr/03_hypo_mutations.php). Among these reported *ALPL* mutations, missense mutations are the most prevalent, which account for 71.2%. The remaining mutations are small deletions mutations (11.0%), splicing mutations (4.9%), nonsense mutations (4.6%), small insertions mutations (3.4%), large deletions/duplications mutations (2.9%), insertion/deletion mutations (1.5%) and regulatory mutations (0.2%). Additionally, the great variety of *ALPL* mutations produces numerous combinations of compound heterozygous mutations, which further promotes the diversity of clinical manifestations of HPP. Previously, Michigami et al. analyzed 98 unrelated Japanese HPP patients found that p.Leu520ArgfsX86 (c.1559delT) and p.Phe327Leu (c.979T>C) were the two most common mutations and were usually associated with the perinatal severe and perinatal benign forms of HPP, respectively [11]. The mutation p.Glu191Lys (c.571G>A) was the most frequent mutations in Caucasian HPP patients and was commonly associated with mild forms of HPP [12]. From this aspect, finding more about the correlation between genotypes and phenotypes is crucial for genetic counseling and prognostication. However, to date, studies on the relationship between genotypes and phenotypes in Chinese HPP patients are limited, especially in children.

Therefore, in the present study, to clarify the clinical characteristic and the relationships between genotypes and clinical manifestations of HPP in Chinese children, ten unrelated children diagnosed with different forms of HPP in our hospital were analyzed. Besides, we also reviewed the clinical and mutational features of the previously reported HPP cases of children in China.

## Materials and methods

### Subjects

Ten patients diagnosed with HPP in Beijing Children’s Hospital, Capital Medical University, from 2009 to 2020 were retrospectively studied. All patients were born to non-consanguineous patients and were diagnosed as HPP depending on clinical manifestations and biochemical items. Clinical manifestations, physical examinations, biochemical profiles, and radiological results of each patient were obtained from medical records. Our present study was approved by the Ethics Committee of Beijing Children’s Hospital, Capital Medical University. The written informed consent was obtained from all the patients and their parents before they participating in the study.

### Clinical information

Patient 1 (PA-1) was a 2.5-month-old boy presenting with poor appetite, soft limbs, less activity, cry, and easy to wake up for 2 months. His height was 60.0 cm (25th–50th), and his weight was 5 kg (10th). He showed underweight, low muscular tension, and enlargement of the anterior fontanelle. Biochemistry indexes revealed low serum ALP activity but high serum calcium (Ca) level (Table 2). Serum levels of phosphate (P), intact parathyroid hormone (i-PTH), 25 hydroxyvitamin D3 [25(OH)D3], as well as urinary calcium to creatinine ratio (Ca/Cr), were all in the normal range (Table 1). The X-ray of chest and lower limb long bones demonstrated the decreased bone density of the metaphysis of long bones, distal ribs, and the margin of the irregular bones, the increased distance between epiphysis and metaphysis. Abdominal ultrasound examinations showed calcium deposits in the bilateral renal medulla. He was clinically diagnosed with infantile HPP.

**Table 1:** Clinical and biochemical features of Chinese children with HPP

Disease subtype	No	Gender	Age of onset/diagnosis	Height (cm)	Weight (kg)	Early deciduous tooth loss	Bone deformity	Epilepsies	Nephrocalcinosis
Perinatal lethal	1	M	2 h/2 h	47.0 (3th)	3.56 (50th–75th)	−	+	+	−
	2	M	12 h/12 h	45.0 (< 3th)	2.80 (10th–25th)	−	+	−	−
Infantile	3	M	0.5 m/2.5 m	60.0 (25th–50th)	5 (10th)	−	+	−	+
	4	F	1 m/5 m	63.0 (10th–25th)	4.3 (< 3th)	−	+	−	+
	5	M	2 m/4 m	57.0 (< 3th)	5.0 (< 3th)	−	+	−	+
	6	M	1 m/3 m	53.0 (< 3th)	3.6 (< 3th)	−	+	−	+
	7	M	1 m/3 m	56.0 (< 3th)	4.1 (< 3th)	−	+	−	+
	8	F	1 m/3 m	54.0 (< 3th)	3.7 (< 3th)	−	+	−	+
	9	M	1 d/2 m	< 10th	NA	−	+	+	−
	10	M	1 m/5 m	56.0 (< 3th)	2.59 (< 3th)	−	+	−	−
	11	F	NA/4 m	54.0 (< 10th)	4.23 (< 10th)	−	+	−	+
	12	M	2 m/4 m	58.0 (< 3th)	5.7 (< 3th)	−	+	−	+
Childhood	13	M	1 y/6 y+5 m	NA	22.0 (25th–50th)	−	+	−	−
	14	F	1 y/2 y + 5 m	50th	NA	+	+	−	−
	15	M	1 y/8 y	25th–50th	NA	+	+	−	−
	16	F	8 m/8 y	118 (3th)	21 (10th)	+	+	−	−
	17	M	1 y/15 y	160.5 (3th–10th)	NA	+	+	−	−
	18	F	NA/5 y	50th	50th	+	+	−	−
	19	M	NA/5.5 y	NA	NA	+	+	−	−
	20	M	NA/18 m	NA	NA	+	+	−	−
	21	F	2 y/19 y	137.0 (< 3th)	35.0 (< 3th)	+	+	−	−
	22	M	2 y/8 y	NA	NA	+	+	−	−
Odonto	23	M	12 m/14 m	NA	NA	+	−	−	−
	24	M	NA/1 y + 9 m	93.0 (> 97th)	14.9 (50th–75th)	+	−	−	−
	25	M	11 m/14 m	79.0 (50th–75th)	10 (25th–50th)	+	−	−	−
	26	M	1.5 y/4 y	25th–50th	NA	+	−	−	−
	27	M	1.3 y/2.3 y	95.0 (75th–90th)	14.0 (50th–75th)	+	−	−	−
	28	F	1 y/6 y	111.4 (10th–25th)	19 (25th–50th)	+	−	−	−
	29	M	1 y/2 y	NA	NA	+	−	−	−
	30	M	1 y/16 y	NA	NA	+	−	−	−
	31	M	NA/14 y	NA	NA	+	−	−	−
	32	M	NA/6 y	NA	NA	+	−	−	−
	33	F	NA/18 m	NA	NA	+	−	−	−

Disease subtype	No	Gender	Serum ALP (U/L)	Serum Ca (mmol/L)	Serum P (mmol/L)	Serum 25(OH)D3 (ng/mL)	Serum i-PTH (pg/mL)	Urinary Ca/Cr	Prognosis	References
Perinatal lethal	1	M	< 5 ↓	Normal	Normal	NA	NA	NA	Die	[31]
	2	M	< 5 ↓	2.2	2.78 ↑	NA	33.4	NA	Die	[32]
Infantile	3	M	26 ↓	3.46 ↑	1.92 ↑	19.0	12.8	0.19	NA	PA-1
	4	F	7 ↓	2.80 ↑	1.38	Normal	2.7 ↓	1.25 ↑	Die	PA-2
	5	M	6 ↓	4.22 ↑	1.41	Normal	2.5 ↓	2.95↑	Die	PA-3
	6	M	16 ↓	3.87 ↑	1.41	19.0	< 1 ↓	2.35 ↑	NA	PA-4
	7	M	5 ↓	3.07 ↑	1.23	34.0	< 1 ↓	2.26 ↑	Die	PA-5
	8	F	23 ↓	4.37 ↑	1.31	24.4	< 1 ↓	1.51 ↑	Die	PA-6
	9	M	5 ↓	3.10 ↑	2.10 ↑	NA	2.91 ↓	NA	Die	[5]
	10	M	9 ↓	3.19 ↑	Normal	NA	3.5 ↓	NA	NA	[33]
	11	F	12 ↓	2.90 ↑	1.52	NA	10.36	2.70 ↑	Die	[34]
	12	M	25 ↓	3.33 ↑	1.34	Normal	< 1 ↓	NA	Die	[35]
Childhood	13	M	36 ↓	2.19	2.23 ↑	NA	10	NA	Live	PA-7
	14	F	42 ↓	2.40	2.0 ↑	Normal	2.91 ↓	NA	Live	[5]
	15	M	67 ↓	2.5	1.7	Normal	14.19	NA	Live	[5]
	16	F	6 ↓	2.68	1.96 ↑	30.7	12.6	NA	Live	[6]
	17	M	26 ↓	2.48	1.89 ↑	Normal	8 ↓	NA	Live	[6]
	18	F	61 ↓	2.47	1.96 ↑	26.64	9.67 ↓	NA	Live	[36]
	19	M	27 ↓	NA	NA	NA	NA	NA	Live	[37]
	20	M	38 ↓	NA	NA	NA	NA	NA	Live	[37]
	21	F	6 ↓	2.55	1.33	5.4 ↓	17.62	NA	Live	[38]
	22	M	27 ↓	Normal	Normal	Normal	NA	NA	Live	[39]
Odonto	23	M	31 ↓	2.25	1.79	24.1	37.7	0.33 ↑	Live	PA-8
	24	M	73 ↓	2.54	2.11 ↑	NA	22.4	NA	Live	PA-9
	25	M	11 ↓	2.45	2.00 ↑	Normal	4.0↓	0.84 ↑	Live	PA-10
	26	M	42 ↓	2.2	1.66	Normal	3.27 ↓	NA	Live	[5]
	27	M	16 ↓	NA	Normal	↓	10.9	NA	Live	[40]
	28	F	22 ↓	2.45	2.03 ↑	NA	8.00 ↓	NA	Live	[6]
	29	M	29 ↓	2.77 ↑	2.24 ↑	NA	16.6	NA	Live	[6]
	30	M	17 ↓	2.52	1.82 ↑	Normal	54	NA	Live	[6]
	31	M	41.5 ↓	Normal	2.08 ↑	↓	Normal	NA	Live	[41]
	32	M	43.4 ↓	Normal	1.97	↓	Normal	NA	Live	[41]
	33	F	7 ↓	NA	NA	NA	NA	NA	Live	[37]

*M* male, *F* female, *h* hour, *d* day, *m* month, *y* year, *ALP* alkaline phosphatase, *Ca* calcium, *P* phosphate, *25(OH)D*_*3*_ 25-hydroxyvitamin vitamin D_3_, *i-PTH* intact parathyroid hormone, *Ca/Cr* calcium/creatinine, *Ref* reference, *NA* not applicable, *H* high, *L* low. ↓ represents the value was below the normal range; ↑ represents the value was above the normal range

Urine Ca/Cr and the blood biochemical parameters, including serum ALP, Ca, P, 25(OH)D3 and i-PTH were measured spectrophotometrically using routine assays in the central laboratory of Beijing Children’s Hospital, Capital Medical University. The normal range for serum ALP, Ca, P, 25 (OH) D3, PTH and urinary Ca/Cr were 58–400 U/L, 2.00–2.75 mmol/L, 1.10–1.80 mmol/L, 19.0–57.6 ng/mL, 10–69 pg/mL, 0.00–0.20, respectively

Patient 2 (PA-2) was a 5-month-old girl presenting with poor appetite, failure to gain weight, soft limbs for 4 months. She was 63.0 cm (10th–25th) in height and 4.3 kg (< 3th) in weight. She showed underweight, developmental delay, weak mental response, low muscular tension, rachitic rosary, Harrison groove, craniotabes, and enlargement of the anterior fontanelle. Biochemistry indexes revealed decreased serum ALP and PTH levels, elevated serum Ca and urinary Ca/Cr, and normal serum levels of P and 25(OH)D3 (Table 1). The X-ray of the chest and both carpal bones demonstrated the thin ribs, and decreased density of the ribs, distal ulnar, and radial bones, and also showed multiple low-density lines in the bilateral distal ulnar and radial bones, and the bilateral scapula. Head computed tomography (CT) demonstrated the general widening of cranial sutures and multiple skull osteogenesis imperfecta. Abdominal ultrasound examinations showed diffuse calcium deposits in the medulla of both kidneys. She was clinically diagnosed with infantile HPP.

Patient 3 (PA-3) was a 57.0 cm (< 3th), 5.0 kg (< 3th), 4-month-old boy presenting with feeding difficulties and vomiting for 2 months. He showed underweight, developmental delay, weakness, and enlargement of the anterior fontanelle. Biochemistry indexes revealed decreased serum ALP and i-PTH levels, elevated serum Ca and urinary Ca/Cr, and normal serum levels of P and 25(OH)D3 (Table 1). The X-ray demonstrated uneven long bone density and the widened distance between epiphysis and metaphysics. The spine and skull showed decreased bone density and a thin cranial plate. Chest CT demonstrated the generally decreased bone density and the enlarged soft tissue density at the head of the ribs. Abdominal ultrasound examinations showed diffuse calcium deposits in the medulla of both kidneys. He was clinically diagnosed with infantile HPP.

Patient 4 (PA-4) was a boy, aged 3-month-old, 53.0 cm (< 3th), and 3.6 kg (< 3th), presenting with poor appetite, failure to gain weight, vomiting for 2 months. He showed underweight, developmental delay, weakness, low muscular tension, widely spaced eyes, low ear position, low nasal bridge, and enlarged anterior fontanelle. Biochemistry indexes revealed decreased serum ALP and i-PTH levels, elevated serum Ca and urinary Ca/Cr, and normal serum levels of P and 25(OH)D3 (Table 1). The X-ray demonstrated the uneven bone density and the multiple bone destruction at the metaphysis of long bones. Abdominal ultrasound examinations showed diffuse calcium deposits in the medulla of both kidneys. He was clinically diagnosed with infantile HPP.

Patient 5 (PA-5) was a 3-month-old boy presenting with poor appetite and failure to gain weight for 2 months. His length was 56.0 cm (< 3th) and his weight was 4.1 kg (< 3th). He showed underweight, developmental delay, and enlargement of the anterior fontanelle. Biochemistry indexes revealed decreased serum ALP and i-PTH levels, elevated serum Ca and urinary Ca/Cr, and normal serum levels of P and 25(OH)D3 (Table 1). The X-ray of limbs demonstrated the stubby bones, the unclear boundary of cortex and medulla, the uneven decreased bone density, and the localized lucency shadow in the metaphysis. The X-ray showed decreased bone density and the widening cranial suture, and the cup-like changes in the metaphysis of the left distal radius and ulna. Abdominal ultrasound examinations showed diffuse calcium deposits in the medulla of both kidneys. He was clinically diagnosed with infantile HPP.

Patient 6 (PA-6) was a 54.0 cm (< 3th), 3.7 kg (< 3th), 3-month-old girl presenting with feeding difficulties, and failure to gain weight for 2 months. She showed underweight, developmental delay, and enlargement of the anterior fontanelle. Biochemistry indexes revealed decreased serum ALP and i-PTH levels, elevated serum Ca and urinary Ca/Cr, and normal serum levels of P and 25(OH)D3 (Table 1). The X-ray of the chest demonstrated the thin ribs and the uneven decreased bone density of the ribs. The X-ray of both lower extremities showed the lightly curved tibia, the blurred metaphysis, and the uneven decreased bone density. Abdominal ultrasound examinations showed diffuse calcium deposits in the medulla of both kidneys. She was clinically diagnosed with infantile HPP.

Patient 7 (PA-7) was a 6-year and 5-month-old boy presenting with intermittent claudication for more than 5 years. His body weight was 22 kg (25th–50th). He showed claudication, swelling, and tenderness of the knee joint. Biochemistry indexes revealed decreased serum ALP levels, elevated serum P levels, and normal serum levels of Ca and i-PTH (Table 1). The X-ray of knees showed the multiple bone destruction at the metaphysis and epiphysis of both knees, accompanied by soft tissue swelling. The X-ray of the pelvis showed the small low-density shadow in the bilateral ischia, which suggested the bone destruction of the ischia. The X-ray scans of his hands and ankle joints showed no abnormal signs. Chest and sacroiliac joint CT as well as the abdominal ultrasound examinations also showed no abnormalities. He was clinically diagnosed with childhood HPP.

Patient 8 was a 1 year and 2 months old boy with early deciduous teeth loss. He had a premature loss of deciduous teeth 2 months after the eruption. When he came to our clinic, he had lost two teeth. No bone deformities were found on physical examination. Biochemistry indexes revealed decreased serum ALP levels, elevated urinary Ca/Cr, and normal serum levels of Ca, P, 25(OH)D3, and i-PTH (Table 1). He was diagnosed with odonto-HPP.

Patient 9 was a 1 year and 9 months old boy with the premature loss of the deciduous teeth. He had lost one tooth when he came to our clinic. No bone deformities were found on physical examination. Biochemistry indexes revealed decreased serum ALP levels, elevated serum P levels, and normal serum levels of Ca and i-PTH (Table 1). The X-ray scans of his chest, left carpal bone, knee joint, and hip joint showed no abnormal findings. He was diagnosed with odonto-HPP.

Patient 10 was a 1 year and 2 months old boy with the premature loss of the deciduous teeth. He had lost two teeth when he was 11-month-old. No bone deformities were found on physical examination. Biochemistry indexes revealed decreased serum ALP and i-PTH levels, elevated serum P and urinary Ca/Cr, and normal serum levels of Ca and 25(OH)D3 (Table 1). The X-ray scans of his chest, left carpal bone, skull, spine, and pelvis revealed no abnormality. There were no abnormal signs of abdominal ultrasound examinations. He was diagnosed with odonto-HPP.

### *ALPL* gene mutation analysis

Genomic DNA of patients 2–10 and their relatives available was extracted from peripheral venous blood and sequenced by the Sanger method to screen for genetic variations of the *ALPL* gene. Sequences generated from samples were compared with the published *ALPL* sequence (accession no: DNA: NG_008940.1, cDNA: NM_000478.6, protein: NP_000469.3). The variants were recognized as mutations when they were not found in dbSNP (dbSNP, https://www.ncbi.nlm.nih.gov/snp/), in Exome Sequencing Project (http://evs.gs.washington.edu/EVS/), and in the 1000 Genomes Project database (https://www.ncbi.nlm.nih.gov/variation/tools/1000genomes/). Three bioinformatics tools PolyPhen-2 (http://genetics.bwh.harvard.edu/pph), SIFT (http://sift.jcvi.org/), and MutationTaster (http://www.mutationtaster.org/) were used to predict the effects of identified mutations on protein structure and function.

### Case review

To provide a comprehensive overview of the reported HPP cases in Chinese children, from March 2005 to April 2019, all publications regarding Chinese children HPP cases in the PubMed database (https://www.ncbi.nlm.nih.gov/pubmed) were reviewed. Additionally, the clinical, biochemical, and molecular characteristics of all previously reported cases were also reviewed.

### Statistical analysis

Statistical analysis was conducted using SPSS for Windows version 20.0 (SPSS Inc., Chicago, IL). Results were presented as median (25th, 75th percentiles), and were compared by Mann–Whitney U-test. Chi-square test was used to assess differences in proportions. Graphs were plotted using GraphPad Prism version 7.0 (San Diego, CA) and Illustrator for Biological Sequences (IBS, Wuhan, China). *P* values < 0.05 were considered statistically significant.

## Results

### Clinical features

Ten patients in our center were included in this study, and ten published papers with another twenty-three patients were also reviewed. 15/23 (65.22%) cases of the reported HPP were within the past three years, indicating an increasing trend of the awareness, detection, and diagnosis of HPP (Additional file 1: Figure S1). In total, thirty-three Chinese children with HPP were identified, and the clinical features of all patients were summarized in Table 1. Two patients were classified as perinatal lethal HPP (6.1%), ten as infantile HPP (30.3%), ten as childhood HPP (30.3%), and eleven patients as odonto-HPP (33.3%). However, there was no report on perinatal benign HPP in Chinese children. The male-to-female ratio was 24:9. The average onset age was 0.69 years (ranged from 2 h after birth to 14 years), while the average diagnosis age was 3.87 years (ranged from 2 h after birth to 19 years). Twenty-three patients (70%) had delayed diagnosis, which was more serious in the forms of childhood and odonto-HPP. Eighteen (54.5%) patients showed short stature, and fifteen (45.4%) patients showed low body weight, both of which were more common in patients with infantile HPP. Nine out of ten (90.0%) patients with childhood HPP, and all patients with odonto-HPP presented early deciduous tooth loss. All patients had bone deformities. Epilepsies were observed in one patient with perinatal lethal HPP and one patient with infantile HPP. Calcium deposits in the medulla of both kidneys were reported in eight out of ten (80.0%) patients with infantile HPP. Except for three cases lost to follow-up, all patients with perinatal lethal/infantile HPP have died.

### Biochemical parameters

**Fig. 1:**
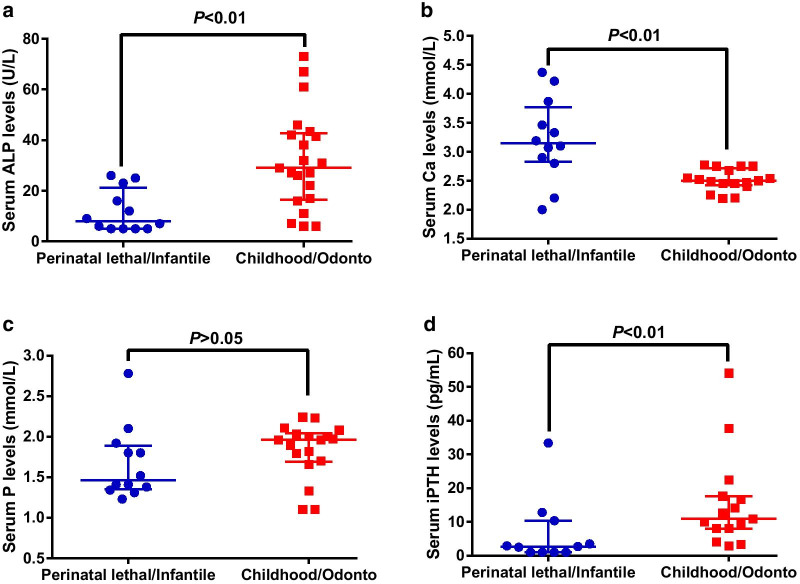
Comparison of serum ALP (**a**), Ca (**b**), P (**c**) and i-PTH (**d**) levels between patients with perinatal lethal/infantile HPP and childhood/odonto HPP. Data were shown as median with interquartile. Abbreviations are as follows: *ALP* alkaline phosphatase, *Ca* calcium, *P* phosphate, *i-PTH* intact parathyroid hormone

The biochemical parameters of thirty-three patients were also shown in Table 1. All patients showed decreased serum ALP levels. Besides, in comparison with patients with the childhood/odonto forms of HPP, serum ALP levels were significantly decreased in patients with perinatal lethal/infantile forms of HPP (*P* < 0.01) (Fig. 1a). All patients with infantile HPP showed elevated serum Ca levels, and serum Ca levels were elevated in patients with perinatal lethal/infantile forms of HPP when compared with those with the childhood/odonto forms of HPP (*P* < 0.01) (Fig. 1b). Fifteen patients showed elevated serum P levels (45.4%). No significant difference was found in serum P levels between patients with childhood/odonto and perinatal lethal/infantile forms of HPP (*P* > 0.05) (Fig. 1c). Only three (0.09%) patients showed decreased serum 25(OH)D3 levels. Eight out of ten (80.0%) patients with infantile HPP had decreased i-PTH levels, and serum i-PTH levels were decreased in patients with perinatal lethal/infantile forms of HPP in comparison with those with the childhood/odonto forms of HPP (*P* < 0.01) (Fig. 1d). Besides, the elevated random urinary Ca/Cr ratios were observed in six (60.0%) infantile HPP patients.

### *ALPL* gene mutations

**Fig. 2:**
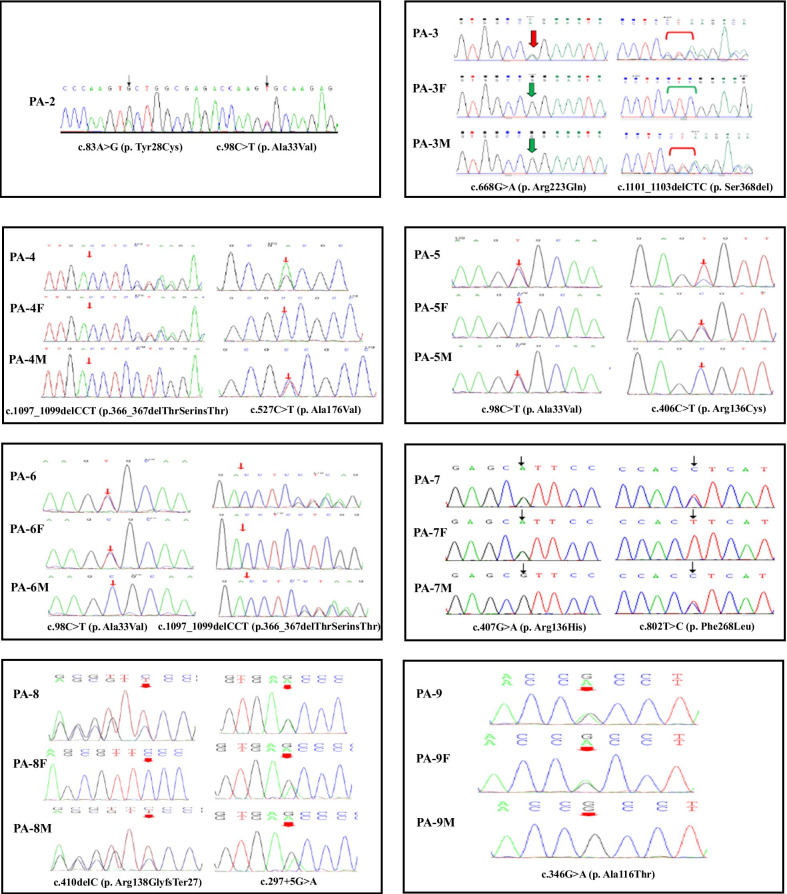
Genetic analysis of *ALPL* mutations in patients 2–9 and their parents. The arrow indicates the position of the mutation

Mutational analysis of the *ALPL* gene were performed in all patients involved in our present study, except for one patient with infantile HPP. The sequences of *ALPL* mutations identified in patients 2–9 were shown in Fig. 2, and the sequences identified in patient 10 were shown in our previous published paper [13]. Fourteen mutations were identified in our present study. Nine mutations in PA-2–7 and PA-9–10 were previously identified (p.Tyr28Cys, p.Ala33Val, p.Arg223Gln, p.Ser368del, p.366_367delThrSerinsThr, p. Arg136Cys, p.Arg136His, p.Ala116Thr, p.Tyr388His). The remaining five mutations were novel: two missense mutations (p.Ala176Val, p.Phe268Leu) that found in PA-4 and PA-7, two frameshift mutations (p.Arg138GlyfsTer27, p.Leu511Profs*272) that found in PA-8 and PA-10, and one splice junction alteration (c.297+5G>A) that found in PA-8. According to the ACMG/AMP variant interpretation guidelines, the novel mutations were classified as likely pathogenic (p. Arg138GlyfsTer27, p. Ala176Val, p. Leu511Profs*272) and uncertain (p. Phe268Leu, c.297+5G>A), respectively.

Additionally, the genetic features of all HPP patients were also summarized. As shown in Table 2, twenty-five (80.6%) patients were compound heterozygous, four (12.9%) were heterozygous, and two (6.5%) were homozygous for mutations in the *ALPL* gene.

**Table 2:** *ALPL* gene mutations of Chinese children with HPP

Disease subtype	No	Status	Type	DNA	Protein	Exon/intron	Source	References
Perinatal lethal	1	Compound heterozygous	M	c.406C>T	p. Arg136Cys	E5	Father	[31]
			M	c.461C>T	p. Ala154Val	E5	Mother	
	2	Compound heterozygous	F	c.650delTinsCTAA	p.217delValinsAlaLys	E7	Mother	[32]
			F	c.984_986delCTT	p. Phe328del	E9	Father	
Infantile	3	NA	NA	NA	NA	NA	NA	PA-1
	4	Compound heterozygous	M	c.83A>G	p. Tyr28Cys	E3	NA	PA-2
			M	c.98C>T	p. Ala33Val	E3	NA	
	5	Compound heterozygous	M	c.668G>A	p. Arg223Gln	E7	de novo	PA-3
			F	c.1101_1103delCTC	p. Ser368del	E10	Mother	
	6	Compound heterozygous	F	c.1097_1099delCCT	p.366_367delThrSerinsThr	E10	Father	PA-4
			M	**c.527C**>**T**	**p. Ala176Val**	E6	Mother	
	7	Compound heterozygous	M	c.98C>T	p. Ala33Val	E3	Mother	PA-5
			M	c.406C>T	p. Arg136Cys	E5	Father	
	8	Compound heterozygous	M	c.98C>T	p. Ala33Val	E3	Father	PA-6
			F	c.1097_1099delCCT	p.366_367delThrSerinsThr	E10	Mother	
	9	Homozygous	M	c.359G>C	p. Gly120Ala	E5	NA	[5]
	10	Compound heterozygous	F	c.228delG	p. Gln76Hisfs*46	E4	Mother	[33]
			M	c.407G>A	p. Arg136His	E5	Father	
	11	NA	NA	NA	NA	NA	NA	[34]
	12	Compound heterozygous	M	c.814C>T	p. Arg272Cys	E7	Father	[35]
			F	c.1101_1103delCTC	p. Ser368del	E9	Mother	
Childhood	13	Compound heterozygous	M	c.407G>A	p. Arg136His	E5	Father	PA-7
			M	**c.802T**>**C**	**p. Phe268Leu**	E8	Mother	
	14	Compound heterozygous	M	c.212G>A	p. Arg71His	E4	Father	[5]
			M	c.571G>A	p. Glu191Lys	E6	Mother	
	15	Compound heterozygous	M	c.203C>T	p. Thr68Met	E4	Mother	[5]
			M	c.571G>A	p. Glu191Lys	E6	Father	
	16	Heterozygous	M	c. 1162T>C	p. Tyr388His	E10	Mother	[6]
	17	Heterozygous	F	c.412_413insC	p. Arg138Profs45x	E5	Mother	[6]
	18	Compound heterozygous	M	c.1183A>G	p. Ile395Val	E10	Father	[36]
			M	c.85T>C	p. Trp29Arg	E3	Mother	
	19	Compound heterozygous	M	c.407G>A	p. Arg136His	E5	Father	[37]
			M	c.1166C>A	p. Thr389Asn	E10	Mother	
	20	Compound heterozygous	M	c.331G>A	p. Ala111Thr	E5	Mother	[37]
			M	c.655A>G	p. Met219Val	E7	Father	
	21	Homozygous	SJA	c.298-1G>A	–	IVS4	NA	[38]
	22	Heterozygous	M	c.251A>T	p. Glu84Val	E4	Mother	[39]
Odonto	23	Compound heterozygous	F	**c.410delC**	**p. Arg138GlyfsTer27**	E5	Mother	PA-8
			SJA	**c.297 + 5G**>**A**	–	IVS4	Father	
	24	Heterozygous	M	c.346G>A	p. Ala116Thr	E5	Father	PA-9
	25	Compound heterozygous	M	c. 1162T>C	p. Tyr388His	E10	de novo	PA-10
			F	**c. 1532insC**	p. Leu511Profs*272	E12	Mother	
	26	Compound heterozygous	M	c.979T>C	p. Phe327Leu	E9	Mother	[5]
			F	c.1017dupG	p. His340Alafs	E10	Father	
	27	Compound heterozygous	M	c.542C>T	p. Ser181Leu	E6	Mother	[40]
			M	c.1287G>T	p. Glu429Asp	E11	Father	
	28	Compound heterozygous	M	c.422C>A	p. Thr141Asn	E5	NA	[6]
			M	c.1489T>A	p. Cys497Ser	E12	NA	
	29	Compound heterozygous	M	c.422C>A	p. Thr141Asn	E5	NA	[6]
			M	c.1489T>A	p. Cys497Ser	E12	NA	
	30	Compound heterozygous	M	c.406C>T	p. Arg136Cys	E5	NA	[6]
			M	c.407G>A	p. Arg136His	E5	NA	
	31	Compound heterozygous	M	c.787T>C	p. Tyr263His	E7	Father and mother	[41]
			R	c.-92C>T	–	E2	Mother	
	32	Compound heterozygous	M	c.787T>C	p. Tyr263His	E7	Father and mother	[41]
			R	c.-92C>T	–	E2	Mother	
	33	Compound heterozygous	M	c.82T>G	p. Tyr28Asp	E3	Father	[37]
			M	c. 1162T>C	p. Tyr388His	E10	Mother	

Bold type indicates the novel mutations identified in our present study

*NA* not available, *M* Missense, *F* Frameshift, *SJA* splice junction alteration

**Fig. 3:**
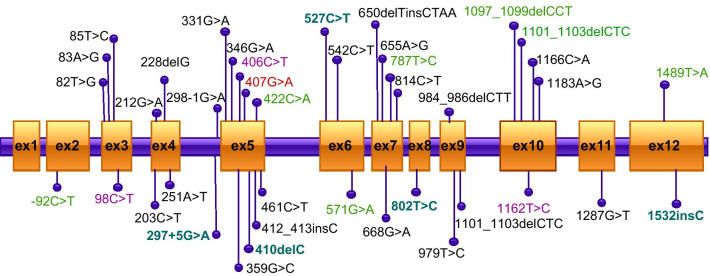
The *ALPL* mutational spectrum in all patients with HPP. Mutations repeated four times were shown in red color. Mutations repeated three times were shown in purple color. Mutations repeated two times were shown in green color. Mutations repeated only one time were shown in black color. Novel mutations that identified in the present study were bold and colored in blue

**Fig. 4:**
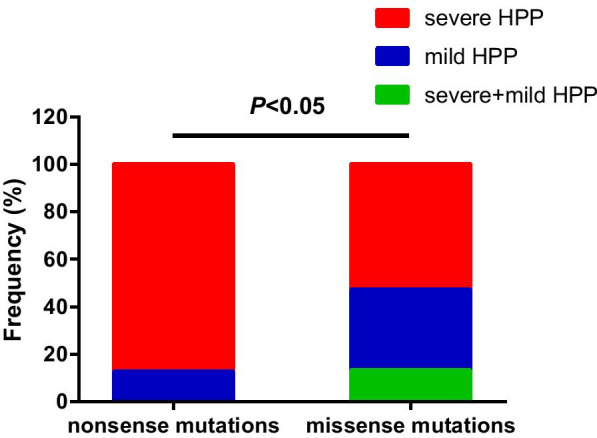
Comparison of phenotypes between nonsense and missense *ALPL* mutations. Abbreviations are as follows: *HPP* hypophosphatasia. Mutations presented only as severe forms of HPP were shown in red color. Mutations presented only as mild forms of HPP were shown in blue color. Mutations presented as severe/mild forms of HPP were shown in green color

Missense variants (n = 28, 70.0%) and frameshift mutations (n = 9, 22.5%) were responsible for the majority of the allelic alterations, whereas splice junction alterations (n = 2, 5.0%) and regulatory mutations (n = 1, 2.5%) were rare. The most prevalent variants were p. Arg136His (n = 4 alleles, 7.14%), followed by p. Arg136Cys (n = 3 alleles, 5.36%), p. Ala33Val (n = 3 alleles, 5.36%), and p. Tyr388His (n = 3 alleles, 5.36%). As shown in Fig. 3, most mutations of *ALPL* were located in exons 5, 7, 10, and 3. It is worth noting that in the present study, subjects (patients 16, 17, 22, 24) carrying single heterozygous mutation showed milder phenotypes of HPP. Since no nonsense mutations were found in our study, we analyzed phenotypic differences between nonsense and missense variants, which have been reported in the Tissue Nonspecific Alkaline Phosphatase Gene Mutations Database (http://www.sesep.uvsq.fr/03_hypo_mutations.php). Compared to missense mutations, nonsense mutations were associated with a severer phenotype (*P* < 0.05) (Fig. 4).

## Discussion

HPP is a rare inherited metabolic disorder. Currently, the prevalence of severe HPP has been estimated to be between 1/100,000 and 1/300,000, whereas mild forms of HPP are more common than severe forms (estimated at 1/6370 in Europe) [7]. China has a large territory and the largest population in the world. So far, only 23 children with HPP were reported in China, and 15 cases were reported within the past 3 years. This suggested that the actual number of pediatric HPP cases in China may be underestimated. Here we described the clinical and genetic features of the 23 reported HPP cases as well as 10 HPP cases diagnosed in our hospital to improve the awareness of pediatric HPP in China.

We found that the most common form of HPP in Chinese children was odonto-HPP, followed by infantile and childhood HPP. Only two patients with perinatal lethal HPP, and no patient was documented as perinatal benign HPP, which indicated the low rate of perinatal HPP in China. In contrast to our results, Michigami et al. analyzed 98 unrelated Japanese HPP patients found that perinatal lethal was the most frequent form of HPP in Japan, followed by the perinatal benign form [11]. Diagnostic delay is common among patients with HPP [14]. Vogt et al. conducted a retrospective review of 50 pediatric HPP patients in Germany found the obvious diagnostic delay in infantile (12 months) and childhood HPP (22.5 months) [15]. Similar findings were also identified in our present study. The average age at onset was 0.69 years, while the average age at clinical diagnosis was 3.87 years, indicating a significant delay in diagnosis in China. Additionally, the diagnostic delay was noted in infantile HPP and even obvious in childhood HPP. There may be several reasons for the diagnostic delay, including low awareness, heterogeneity of clinical manifestations, and lack of routine testing of HPP.

As previously described, the clinical spectrum of HPP was highly variable, the main clinical manifestations of the pediatric Chinese patients were in line with those documented in other studies [2, 3, 14]. Failure to thrive was one of the most common manifestations of pediatric HPP patients [16], in the current study, we found that 18 (54.5%) patients showed short stature, and 15 (45.4%) patients showed low body weight, both of which were more common in patients with infantile HPP. Therefore, serum ALP levels should be part of a routine accessory examination in infants and children with problems in gaining weight, growth retardation, and short stature. Similar to other studies [17], we found a higher proportion of males (n = 24, 72.73% approximately) than females (n = 9, 27.27% approximately) diagnosed with pediatric HPP. Indeed, previous studies performed by Grimberga et al. pointed out that compared with boys, girls gained less evaluation for short stature [18]. Thus, the greater parental concern about short stature in sons versus daughters may be one of the explanations for the higher proportion of males in pediatric HPP patients. It is worth noting that contrary to the pediatric HPP, a higher proportion of females than males was observed in adult HPP [14, 19–21]. Considering the small number of pediatric HPP patients diagnosed in our country, the sex differences observed in the current study still need to be verified in further studies.

Serum ALP levels were decreased in all HPP patients. From the literature, it is known that serum ALP levels seem to be correlated with the disease severity [15, 22, 23]. Consistent with this, our present study conducted in the Chinese pediatric HPP patients also demonstrated significantly decreased serum ALP levels in patients with perinatal lethal/infantile forms of HPP than those with the childhood/odonto forms of HPP. Interestingly, we observed that serum Ca and i-PTH levels were also correlated with disease severity. Serum Ca levels were elevated, while serum i-PTH levels were decreased in patients with perinatal lethal/infantile forms of HPP in comparison with those with childhood/odonto forms of HPP. In support of our results, previous studies have shown that severe hypercalcemia was common, and circulating PTH levels were physiologically suppressed in infantile HPP [2, 24]. However, in childhood HPP, hypercalcemia was less common and serum PTH levels were usually within the normal range [2]. Our findings together with others [25] supported that serum Ca and PTH could provide important complementary diagnostic information, especially for patients with severe forms of HPP.

Genetic detection is a crucial final step in the diagnosis of HPP. Consistent with international research findings, missense mutations were the most common mutations identified in this study, and exon 5 was the predominantly affected exon. The variety of missense mutations resulted in highly variable clinical expressivity and a large number of compound heterozygous genotypes. Also consistent with other results [15], compound heterozygous mutations were far more common than heterozygous and homozygous mutations. Five novel mutations, including p.Arg138GlyfsTer27, p.Leu511Profs*272, p.Ala176Val, p.Phe268Leu, and c.297+5G>A were found in this study. Although we did not conduct functional in vitro studies of the effects of these mutations, evidence from bioinformatics analysis supported the hypothesis that these mutations were harmful. Three of the identified mutations (p.Arg138GlyfsTer27, p.Leu511 Profs*272, c.297+5G>A) could disrupt the protein structure, result in forming a truncated protein or causing a frame-shift, or loss of protein function by changing splice sites, respectively. For the other two missense mutations we identified (p.Ala176Val, p.Phe268Leu): p.Ala176Val was located in the active site valley, and p.Phe268Leu was located in the calcium-binding site. There are five crucial regions identified in TNSALP, including the active site, the active site valley, the homodimer interface, the crown domain, and the calcium-binding site, and mutations that alter residues at these sites may induce dysfunction of the protein and thus cause the HPP [6, 26, 27]. It is worth noting that our study found obvious differences in the spectrum of *ALPL* mutations between Chinese individuals and other countries. For example, p.Leu520ArgfsX86 and p.Asp378Val were the most common mutations in Japan [11] and the USA [17], respectively, while no report has been found in Chinese HPP patients. p.Glu191Lys was known to occur with a high frequency (up to 55%) in HPP patients with European ancestry [28]. However, it has been reported only twice in the Chinese population [5]. These results indicated that a Chinese-specific screening panel may be warranted for the diagnose of Chinese HPP patients. To date, the relationship between genotypes and phenotypes in Chinese pediatric HPP patients with *ALPL* mutations remains unclear. Based on the Tissue Nonspecific Alkaline Phosphatase Gene Mutations Database, we found that nonsense mutations were associated with a severe phenotype in comparison with missense mutations. However, no nonsense mutation was found in pediatric HPP patients in China. Previous studies have shown that patients with a single heterozygous mutation usually presented with mild forms of HPP [14]. The same was seen in our present study, subjects (patients 16, 17, 22, 24) carrying single missense mutation or frameshift mutation showed milder phenotypes of HPP. The number of HPP patients in this study was relatively small considering the large population of China. As the number of HPP patients increases, the genotype–phenotype correlation may be clear in the future.

Our study also showed high mortality in patients with severe forms of HPP. Except for three cases lost to follow-up, all patients with severe forms of HPP (perinatal lethal/infantile HPP) have died. Previous studies have shown that approximately 50% of cases with infantile HPP were predicted to die [2], and if patients manifested with chest deformity, respiratory difficulties, or vitamin B6-dependent seizures before 6 months of age, the mortality was significantly higher [29]. Until recently, the management of HPP in China has been symptomatic and supportive only. In 2015, bone-targeted enzyme-replacement therapy (asfotase alfa) was approved in Japan, and then in Canada, in the European Union, and in the United States to treat pediatric-onset HPP [2]. This enzyme has produced beneficial effects not only in bones but also in other organs, including the lungs and muscle [30]. While asfotase alfa seems very promising, it is not approved in China yet, and many questions regarding its long-term effects and the potential secondary adverse effects remain to be solved in the future.

There are still some limitations in the present study. First, due to its retrospective design, not all clinical or biochemical data were available from all patients. Further follow-up study will be conducted in order to explore the long-term prognosis of patients with mild forms of HPP. Second, although serum ALP, Ca and i-PTH levels were associated with the disease severity, the best cutoffs were unclear. Additionally, PLP and PEA are useful parameters for the diagnostic of HPP and serum PLP levels have been reported to be correlated with the disease severity [12]. However, due to the limited medical equipment in our hospital, PLP and PEA levels were not detected in the present study. Third, in vitro functional experiments will be needed to characterize the function of the novel mutants. Fourth, the number of patients was not big enough due to low awareness. Future research is required to reveal the phenotype-genotype correlations.

In conclusion, our study shows that HPP remains a complex disease with a variable phenotype. Although the number of HPP cases has increased reported in recent years, the incidence of HPP may still be underestimated because of a lack of disease awareness. Diagnosis is often delayed in particular in patients with mild forms of HPP. The mutation spectrum of *ALPL* in China is quite different from those in other countries. This is the first time to summarize the clinical and genetic characteristics of pediatric HPP patients in China. In the future, further studies in larger cohorts should be conducted to evaluate the phenotype-genotype association in Chinese HPP patients to improve and shorten the diagnosis of HPP.

## Supplementary Information

**Additional file 1: Figure S1.** The number of HPP cases diagnosed in Chinese children from 2005 to 2019. Abbreviations are as follows: *HPP* hypophosphatasia.

## Data Availability

All data generated and analyzed during this study are included in this published article and are available from the corresponding author upon reasonable request.

## Abbreviations

ALP: Alkaline phosphatase
Ca: Calcium
Ca/Cr: Calcium to creatinine ratio
CT: Computed tomography
F: Female
HPP: Hypophosphatasia
25(OH)D3: 25 Hydroxyvitamin D3
i-PTH: Intact parathyroid hormone
M: Male
P: Phosphate
PEA: Phosphoethanolamine
PLP: Pyridoxal-5-phosphate
PPi: Inorganic pyrophosphate
SJA: Splice junction alteration
TNSALP: Tissue-nonspecific isozyme of alkaline phosphatase
