# Supplementary figures and images for: Clinical and genetic characteristics of hypophosphatasia in Chinese children
Source: Orphanet J Rare Dis. 2021 Apr 7;16:159. doi: 10.1186/s13023-021-01798-1 (PMC8028151; doi:10.1186/s13023-021-01798-1)

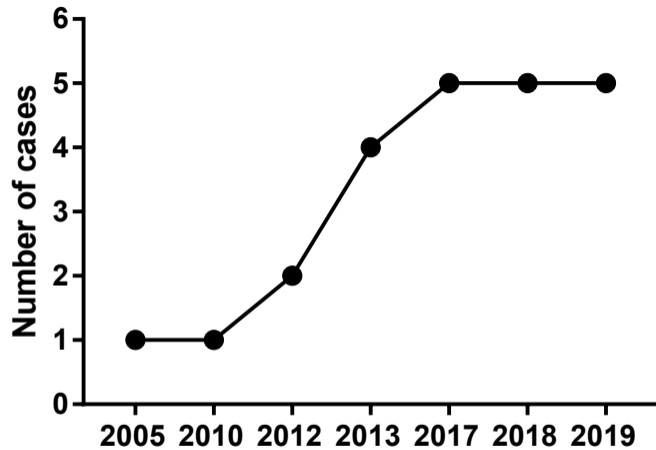

**Supplementary Figure 1. The number of HPP cases diagnosed in Chinese children from 2005 to 2019.**

Supplement: Supplementary file 1 — Additional file 1: Figure S1. The number of HPP cases diagnosed in Chinese children from 2005 to 2019. Abbreviations are as follows: HPP hypophosphatasia. [file 13023_2021_1798_MOESM1_ESM.pdf]
